# Supplementary material for: Multi-label classification of symptom terms from free-text bilingual adverse drug reaction reports using natural language processing
Source: PLoS One. 2022 Aug 4;17(8):e0270595. doi: 10.1371/journal.pone.0270595 (PMC9352066; doi:10.1371/journal.pone.0270595)
Supplement: S1 Appendix — (PDF) [file pone.0270595.s001.pdf]

S1 Appendix. Naive Bayes - Support Vector Machine (NB-SVM)

Given that a text document d with n words is represented as a bag of unsorted words $w_i \in \{w_1, ..., w_n\}$. Under the Naive Bayes assumption, the probability of a class $c \in C$ given the document d is:

$$P(c|d) = \frac{P(d|c)P(c)}{P(d)} = \frac{P(w_1, ..., w_n|c)P(c)}{P(d)} = \frac{P(w_1|c)\cdots P(w_n|c)P(c)}{P(d)}. \tag{1}$$

$P(d)$ can be conveniently dropped as the same probability $P(d)$ is applied for all classes. $P(c)$ is a prior probability of the class c which is the ratio of the number of documents in the class c, N_c, to the number of documents in all classes, N_{all}. $P(w_i|c)$ is a likelihood of the word w_i given the class c which is the ratio of the number of occurrences of the word w_i appears in all documents of the class c to the number of occurrences of all words W that appear in all documents the of class c. With the Laplace smoothing to prevent zero probabilities for infrequently occurring words, $P(c)$ and $P(w_i|c)$ can be expressed as:

$$P(c) = \frac{N_c}{N_{\text{all}}}, \tag{2}$$

$$P(w_i|c) = \frac{\text{Count}(w_i, c) + 1}{\sum_{w \in W} \big(\text{Count}(w, c) + 1\big)}. \tag{3}$$

The class that is chosen by the Naive Bayes classifier is the class, $\hat{c}$, that has the maximum posterior probability given the document. For language modelling, Naive Bayes computations is normally performed in a log space to avoid arithmetic underflow. Thus, the equation can be representation as a linear combination of input features:

$$\hat{c} = \underset{c \in C}{\text{argmax}} \; \log\big(P(w_1|c)\cdots P(w_n|c)P(c)\big) \tag{4}$$

$$= \underset{c \in C}{\text{argmax}} \; \sum_{i=1}^{n} \log\big(P(w_i|c)\big) + \log\big(P(c)\big). \tag{5}$$

Instead of searching for the class with the highest posterior probability, SVM can utilizes NB feature values for text classification. SVM can learn to directly assign each weight to NB features, leading to better discrimination ability. Previous studies suggest that NB performs better on short documents, while SVM performs better on longer documents than NB [9].

Let $\mathbf{x}$ denote NB feature sets and $\mathbf{w}^c$ and b^c are the normal vector and bias of the linear SVM hyperplane for the class c. Thus, an SVM classifier can be formulated as

$$f_c(\mathbf{x}) = \text{sign}\big(\mathbf{w}_c^T \mathbf{x} + b_c). \tag{6}$$

In multi-label classification, we ideally want the probabilities to be large for the correct classes and small for the incorrect classes. This can be formulated with SVM by minimizing the following L2-regularized L2-loss optimization function [9]:

$$L(\mathbf{w}_c, b_c) = \frac{1}{2}\mathbf{w}_c^T \mathbf{w}_c + \lambda \sum_i \max\big(0, 1 - y^{(i)}(\mathbf{w}_c^T \mathbf{x}^{(i)} + b_c))\big)^2 \tag{7}$$

where $\lambda > 0$ is a penalty parameter.
