# Supplementary material for: Multi-label classification of symptom terms from free-text bilingual adverse drug reaction reports using natural language processing
Source: PLoS One. 2022 Aug 4;17(8):e0270595. doi: 10.1371/journal.pone.0270595 (PMC9352066; doi:10.1371/journal.pone.0270595)
Supplement: S2 Appendix — (PDF) [file pone.0270595.s002.pdf]

S2 Appendix. Evaluation Metrics.

Given that D is a multi-label dataset of n samples (x_i, Y_i), $1 \leq i \leq n$ and $(x_i \in \mathbf{x}, Y_i \in \mathbf{Y} = \{0,1\}^k)$ with a set of class C in which $|C| = k$. Let f be a multi-label classifier and $Z_i = f(x_i) = \{0,1\}^k$ be a set of class membership predicted by the model

f for the sample x_i. Exact match ratio computes the proportion of fully correctly classified samples to all samples:

$$\text{Exact Match Ratio} = \frac{1}{n}\sum_{i=1}^{n} I(Y_i = Z_i) \tag{8}$$

where I is the indicator function in which $I(A) = 1$ is given an event A is true and $I(A) = 1$ if an event A is false. This metric could be considered harsh, as it disregards completely incorrect and partially correct predictions.

Accuracy computes an average of the proportion of correctly predicted labels to all predicted and ground truth labels; therefore, it takes partially correctness into account:

$$\text{Accuracy} = \frac{1}{n}\sum_{i=1}^{n} \frac{|Y_i \cap Z_i|}{|Y_i \cup Z_i|}. \tag{9}$$

Hamming loss indicates how often on average a class label is incorrectly predicted. Both prediction error (an incorrect label is predicted) and missing error (a relevant label is not predicted) are considered. This metric is normalized to the total number of classes and the total number of samples:

$$\text{Hamming Loss} = \frac{1}{kn}\sum_{i=1}^{n}\sum_{c=1}^{k}[I(c \in Z_i \wedge c \notin Y_i) + I(c \notin Z_i \wedge c \in Y_i]. \tag{10}$$

Precision measures how accurate the class predictions are. It measures the ratio of correctly predicted labels to all ground truth labels, averaged over all samples:

$$\text{Precision} = \frac{1}{n}\sum_{i=1}^{n} \frac{|Y_i \cap Z_i|}{|Z_i|}. \tag{11}$$

Recall measures how good the algorithm correctly predicts class labels. It measures the ratio of correctly predicted labels to all predicted labels, averaged over all samples:

$$\text{Recall} = \frac{1}{n}\sum_{i=1}^{n} \frac{|Y_i \cap Z_i|}{|Y_i|}. \tag{12}$$

F_1-score is a harmonic mean of prediction and recall:

$$\text{F}_1\text{-score} = \frac{2}{\text{Recall}^{-1} + \text{Precision}^{-1}} = \frac{1}{n}\sum_{i=1}^{n} \frac{2|Y_i \cap Z_i|}{|Y_i| + |Z_i|}. \tag{13}$$

Average precision computes per class as a weighted average of the precision values achieved at each classification threshold, with the increase in recall from the previous threshold as the weight:

$$\text{Average Precision} = \sum_{t \in \{0.0,\dots,1.0\}} (\text{Recall}_t - \text{Recall}_{t-1})\text{Precision}_t. \tag{14}$$

where Precision_t and Recall_t are the precision and recall at the threshold t respectively. Mean average precision is the mean of average precision over all classes.

Krippendorff's alpha (α) [32] is a statistical measure of agreement between observers that codes a set of objects of analysis. Given that D_o is the observed disagreement and D_e is the disagreement expected by chance, the alpha of M observers and N objects is given by:

$$\alpha = 1 - \frac{D_o}{D_e} = 1 - (n-1)\frac{\sum_c^N \sum_{k>c}^M o_{ck}\delta^2(c,k)}{\sum_c^N \sum_{k>c}^M n_c n_k \delta^2(c,k)} \tag{15}$$

where o is a coincidence matrix, δ is a metric function of the given data into the two disagreements, and n is the total number of pair elements. The MASI (Measuring Agreement on Set-valued Items) distance, a distance metric for comparing two sets, was used as a metric function. The alpha indicates the extent to which the results can be trusted to represent the true answer. $\alpha = 1$ means perfect reliability.
