# Supplementary material for: Multi-label classification of symptom terms from free-text bilingual adverse drug reaction reports using natural language processing
Source: PLoS One. 2022 Aug 4;17(8):e0270595. doi: 10.1371/journal.pone.0270595 (PMC9352066; doi:10.1371/journal.pone.0270595)
Supplement: Attachment — Submitted filename: Allergy_PLoS_Responses.pdf [file pone.0270595.s004.pdf]

Responses to the Reviewers' Comments

Multi-label classification of symptom terms from free-text bilingual adverse drug reaction reports using natural language processing

Dear Academic Editor and Reviewers,

Thank you for your very thorough review of the manuscript. We greatly appreciate the reviewers' constructive comments, as well as the Academic Editor's decision to allow us to revise and resubmit unclear or overlooked points in our manuscript. We tried to maintain our original motivation and framework for developing the manuscript while correcting and improving the details and unclear statements based on the comments given.

We have addressed all comments as described on the next page. Our responses are as follows and are highlighted in yellow in the revised manuscript. We believe we have greatly improved the article and more clearly explained our findings.

Sitthichok

Sitthichok Chaichulee DPhil
Department of Biomedical Sciences and Biomedical Engineering
Faculty of Medicine, Prince of Songkla University 90110, Thailand.

Reviewer's Responses to Questions

Comments to the Author

1. If the authors have adequately addressed your comments raised in a previous round of review and you feel that this manuscript is now acceptable for publication, you may indicate that here to bypass the “Comments to the Author” section, enter your conflict of interest statement in the “Confidential to Editor” section, and submit your "Accept" recommendation.

Reviewer #2: All comments have been addressed
Reviewer #3: (No Response)

---

2. Is the manuscript technically sound, and do the data support the conclusions?

The manuscript must describe a technically sound piece of scientific research with data that supports the conclusions. Experiments must have been conducted rigorously, with appropriate controls, replication, and sample sizes. The conclusions must be drawn appropriately based on the data presented.

Reviewer #2: Yes
Reviewer #3: Partly

---

3. Has the statistical analysis been performed appropriately and rigorously?

Reviewer #2: Yes
Reviewer #3: N/A

---

4. Have the authors made all data underlying the findings in their manuscript fully available?

The PLOS Data policy requires authors to make all data underlying the findings described in their manuscript fully available without restriction, with rare exception (please refer to the Data Availability Statement in the manuscript PDF file). The data should be provided as part of the manuscript or its supporting information, or deposited to a public repository. For example, in addition to summary statistics, the data points behind means, medians and variance measures should be available. If there are restrictions on publicly sharing data—e.g. participant privacy or use of data from a third party—those must be specified.

Reviewer #2: No
Reviewer #3: No

---

5. Is the manuscript presented in an intelligible fashion and written in standard English?

PLOS ONE does not copyedit accepted manuscripts, so the language in submitted articles must be clear, correct, and unambiguous. Any typographical or grammatical errors should be corrected at revision, so please note any specific errors here.
Reviewer #2: Yes
Reviewer #3: Yes

---

6. Review Comments to the Author

Please use the space provided to explain your answers to the questions above. You may also include additional comments for the author, including concerns about dual publication, research ethics, or publication ethics. (Please upload your review as an attachment if it exceeds 20,000 characters)

Reviewer #2: (No Response)

Reviewer #3:

In this work authors evaluated different NLP algorithms that can encode unstructured ADRs stored in EHRs into institutional symptom terms.

Authors made efforts to revise the first draft of the manuscripts by addressing previous reviewers' comments. However, I have a few concerns on this paper and in my opinion, the manuscript is not in a state to be published. See my comments below

<u>Answer</u>

Thank you for your very thorough review of the manuscript. We appreciate your comments very much. We have improved the article in many aspects and explained our contributions more clearly.

1) The approach leverages existing well-known techniques together to solve an existing problem. It is not clear the key technical contribution of the proposed study. No novelty.

<u>Answer</u>

Our goal is to develop an approach that could help pharmacists to code symptoms. The problem of managing allergy data varies from institution to institution. Previous studies relied on rule-based techniques and the extensive use of data dictionaries. In contexts other than English, this process requires the creation of domain-specific data dictionaries, which is a very time consuming process. In the recent study on Russian ADRs, Lenivtceva and Kopanitsa (2021), developed their own rules and dictionaries which should be reviewed and updated over time. ML/DL offers an approach that does not require data dictionaries, but instead requires lots of data samples for training.

Our novelty relies on the investigation on the applicability of NLP in a domain-specific context, i.e., drug allergy, where there are still gaps in the development of such algorithms, especially in an environment where English is not the native language, as we pointed out in the Introduction section. In our drug allergy context, we generally explain clinical symptoms using free-text descriptions that are a mixture of Thai and English words.

We kindly agree that we have chosen the standard conventional approaches, which are really mature for such implementation. We did, however, provide a thorough comparison of various algorithms, from simple NB-SVM to LSTM-based ULMFiT and newer transformer-based BERT models. We tried two multilingual BERT models: mBERT and XLM-ROBERTa,

one bilingual model: WangchanBERTa and our AllergyROBERTa (pre-trained from scratch on our corpus). Our research reveals that XLM-RoBERTa, which was pre-trained on a very large multilingual general domain corpus, performs well in a clinical setting where a pre-trained LM is not available or data for training a new LM is missing.

We plan to explore NLP applications in other clinical contexts, such as inpatient coding of ICD-10 from discharge summary and clinical phenotype, which indeed require a more complex representation of clinical data.

2) There are many recent studies already published which are using the same idea even with more sophisticated ways of learning text representation. All recent studies are missing in the literature review.

<u>Answer</u>

We have expanded the literature review of previous studies to provide more details about the studies in the Introduction section, Pages 4–5 Lines 144–173.

“Recently, there have been a few studies on the topics [5, 16–20]. Wagholikar et al. [16] developed a rule-based method using the SNOMED-CT (Systematized Nomenclature of Medicine – Clinical Terms) ontology for categorizing free-text chief complaints into symptom groups. The development of such algorithms was challenging due the different ways in which medical narratives were recorded by clinical staff. To deal with variations in clinical notes, Epstein et al. [5] developed an allergy-matching method that incorporates RxNorm, abbreviation, and misspelling terms to identify medication and food allergies from unstructured allergy records. Despite the use of extensive look-up tables, some free-text entries in the test set were not matched by the algorithm. Goss et al. [17] conducted a similar study with a larger set of terms, including SNOMED-CT and ICD-10 (International Classification of Diseases – 10th Revision) terms. Due to the presence of non-standard terms in free-text entries, algorithmic performances still varied. Similarly, Jackson et al. [18] developed SVM models to specifically capture key symptoms of severe mental illness from free-text discharge summaries using disease symptomatology concepts. Some symptoms were missed due to non-standard language usage. In languages other than English, Lenivtceva et al. [19] developed an extensive rule-based model for coding Russian free-text medical records into allergy identification categories that complies the HL7 FHIR (Health Level 7 – Fast Healthcare Interoperability Resources) standard. The method still suffered from lack of generalizability due to the availability of data dictionaries in Russian. Recently, Leiter et al. [20] using deep learning to identify symptoms in patients with congestive heart failure to assess the response of cardiac resynchronization therapy using an expert-labeled set of free-text notes without the representation of medical concepts. The algorithm was, however, inconsistently performed with typographical errors and phrasings that were absent from the training set. The problem could alleviate with a larger set of training samples. Some of the studies presented thus far required the modeling of medical concepts or the extensive use of data dictionaries. Most of the studies were conducted in a monolingual setting. However, in settings where English is not the official language, EHRs are often documented in another language or even a mixture of multiple languages.”

3) Authors should refer to the state-of-the-art methods in Biomedical NLP (bioNLP) (e.g BioBERT and current SOTA BioALBERT). There are many studies which shows that using Biomedical (domain-specific) language models works better than language models trained on general corpus (such as Wikipedia etc). Authors should compare their results and discuss that domain-specific methods

- BioALBERT: A Simple and Effective Pre-trained Language Model for Biomedical Named Entity Recognition
- Benchmarking for Biomedical Natural Language Processing Tasks with a Domain Specific ALBERT
- biobert: a pre-trained biomedical language representation model for biomedical text mining

<u>Answer</u>

We intended to implement current SOTA LMs where available. Unfortunately, all publicly available biomedical and clinical LMs were developed based on an English-only corpus. They cannot be finetuned with our data, which contain a mixture of Thai and English words. For this reason, we chose to use multilingual LMs. Therefore, it is not possible to compare our results with those of biomedical and clinical LMs.

We have added the literature review of biomedical and clinical LMs on Page 4 Lines 114–127.

"Several LMs have been pre-trained on biomedical and clinical corpora. BioBERT was pre-trained over 18 billion words from PubMed abstracts and PubMed Central full-text articles [13]. ClinicalBERT was pre-trained on 2 million de-identified clinical notes from the Beth Israel Deaconess Medical Center [14]. BioALBERT [15] was pre-trained on the datasets used by BioBERT and ClinicalBERT, so the model learns language representation from both biomedical and clinical domain. All three models were reported to outperform the state of the art models pre-trained on general-domain data on most downstream tasks. However, they were all pre-trained with a monolingual English corpus. For the use of BERT models on clinical data, it was recommended to pre-train the model on a private dataset at the practitioner's institution for best results, as clinical notes may vary depending on the clinical setting [14]."

We have expanded the Discussion Section on this issue on Page 16 Lines 510–515.

"Although many studies have shown that biomedical and clinical LMs (such as BioBERT [13], ClinicalBERT [14], and BioALBERT [15]) performed better on downstream clinical tasks than general-domain LMs, all available domain-specific LMs have been pre-trained with a monolingual English corpus only. It is not possible to finetune these LMs with our institutional non-English dataset and compare their performances."

4) The case for the paper is weak. The authors do provide a review of the relevant works however the relevant works are flatly discussed without properly highlighting their weaknesses and establishing the research gaps.

<u>Answer</u>

We have rewritten the Discussion section Page 16–17 Lines 533–575 to highlight the weaknesses of the methods used by the previous studies and establish the research gaps.

"The extraction of clinical symptoms from unstructured clinical texts has been investigated in many studies [5, 16–20] The standardized symptomatic terms resulted from these works have been used for epidemiological research, clinical systems improvement, and healthcare interoperability. It is, however, difficult to make direct comparison to other studies because each study used its own institutional dataset and had its own unique purpose.

Compared with previous studies that used rule-based techniques [5, 16, 17, 19], our techniques were developed without the use of data dictionaries or clinical concept modeling. In the rule-based studies, Wagholikar et al. [16] created a script for mapping various synonyms and acronyms to SNOMED CT concepts. The approach was accurate for specific symptoms such as chest pain but not for more complex groups such as stomach pain and trauma. Epstein et al. [5] implemented an allergy-matching algorithm with Transact-SQL that included seven lookup tables. The algorithm achieved an F1 score of 0.98. The authors pointed out that periodically checking the remaining entries not found by the algorithm and periodically adding terms to the lookup tables could help improve the algorithm. Goss et al. [17] compiled several lists of standard terminologies with a set of rules defined for encoding allergy concepts. The authors noted a need for the algorithm to consider a contextual understanding between allergens and symptoms for best results. Recently, the rule-based pipeline that developed by Lenivtceva et al. [29] employed various techniques to handle misspellings and abbreviations, implemented over 20 regular expression rules, and constructed over four dictionaries of over 2,675 terms to describe allergy categories in Russian. Thanks to the complicated and specialized approach, the authors reported very good performance with F1-Score of 0.90–0.96 for allergy categorization. Implementing the rule-based approach could be time-consuming and tedious, but it is not necessary to have a well-curated large dataset.

The ML/DL approach has been reported to perform well, comparable to or better than rule-based methods, but depends heavily on the quality of the data. In practice, the ML/DL approach requires a well-curated dataset. Classical ML algorithms, such as SVM, have been employed in Jackson et al. [18] that involved human annotators to label clinical documents to in order to create training corpora. The approach can extract symptoms from clinical text with a median F1 score of 0.88 across 46 symptoms. In Leiter et al. [20], a DL algorithm was developed to identify symptoms from clinical notes of patients with congestive heart failure. The algorithm had an F1 score of 0.72 which might be due to a small sample size of only 154 notes.

Our study benefits from the availability of data that have already been labeled by experts over time. Without the need to define explicit rules, the ML/DL approach can reduce the

time and effort required to develop clinical NLP algorithms. In addition, with a large training corpus, the ML/DL approach can learn some degree of noise in the text data, e.g. abbreviations, misspellings, uncertainties, and variations in clinical notes. However, the ML/DL approach may perform poorly on certain underrepresented symptom groups because the algorithm learns the categorization of the text from the data rather than from explicit rules."

5) Some experiment methods need more explanation

Answer

We have revised some experimental methods as per your comments.

6) Finally, various typographical and grammatical errors must be rectified.

Answer

We apologize for the typographical and grammatical errors in the manuscript. We have now corrected the problem.

I would recommend that the authors look through more recent publications on this problem. Establishing novelty of approach over other published work would benefit their work, as well as the manuscript.
